# Anurans or Mice: What is the Best Food Item for Young and Adults of *Bothrops jararacussu* (Lacerda, 1884) in Captivity?

**DOI:** 10.1002/zoo.21904

**Published:** 2025-05-03

**Authors:** Taís Vasques Torrents, Fernanda Dias‐Silva, Luisa Diele‐Viegas, Ana Maria Paulino Telles de Carvalho‐e‐Silva

**Affiliations:** ^1^ Pós‐Graduação em Ciências Biológicas (Biodiversidade Neotropical) Universidade Federal do Estado do Rio de Janeiro Rio de Janeiro Brazil; ^2^ Laboratório Biossistemática de Anfíbios, Departamento de Zoologia, Instituto de Biociências Universidade Federal do Estado do Rio de Janeiro Rio de Janeiro Brazil; ^3^ Museu Nacional, Universidade Federal do Rio de Janeiro Rio de Janeiro Brazil; ^4^ Laboratório de (Bio)Diversidade do Antropoceno, Instituto de Biologia Universidade Federal da Bahia Salvador Brazil; ^5^ Department of Biology University of Mississippi Oxford, MS United States

**Keywords:** bullfrog, captive snake, native anurans, pit viper, snake diets

## Abstract

Good feed management in captivity is essential to animal survival and well‐being. For snakes, most studies focus on the frequency of prey consumption and the types of prey that constitute their diets in the wild. Conversely, there is a notable scarcity of studies regarding their dietary habits in captivity. The prevailing trend involves feeding nearly all snake species exclusively with mice, barring the exceptions found in ophiophagic species. Considering that *Bothrops* species consume different diets in young and adults in the wild, we use *B. jararacussu* as a model to introduce other prey items in captivity. We use a generalized linear model (GLM) to understand if native and exotic anurans can be alternative items in the captivity diet of young and adult individuals. Our glm showed that snakes fed on bullfrog had less weight gain and consequently less growth than those provided with other diet groups. Our experiments indicated that native anurans could be an interesting food alternative to *B. jararacussu* and other Bothrops species with an ontogenetic diet in captivity.

## Graphical abstract

We use *Bothrops jararacussu* as a model to introduce three different prey items (mice, exotic anuran – bullfrog and native anurans) in captivity. Our experiments indicated that native anurans could be an interesting food alternative to *B. jararacussu* and other *Bothrops* species with an ontogenetic diet in captivity.

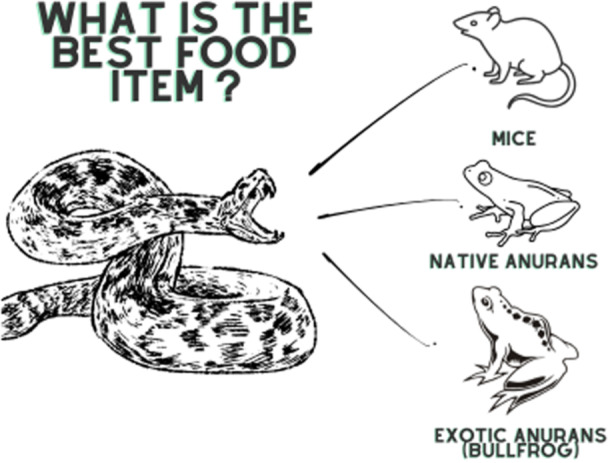

## 1 Introduction

*Bothrops jararacussu* (Serpentes: Viperidae) is an endemic pit viper to South America whose occurrence is restricted to low to intermediate elevations in the Atlantic Forest. Their distribution is known from Argentina and Brazil to Paraguay (Nogueira et al. 2019). Like other pit vipers, *B. jararacussu* is documented as an agent of snakebites in humans. Since 2009, snakebites have been listed within the Neglected Tropical Diseases list by the World Health Organization (WHO), which encourages and prioritizes the facilitation of equitable access to quality antivenom therapies (Squaiella‐Baptistão et al. 2018). In Brazil, estimates point to 31,357 snakebites in 2021, of which 62.9% were caused by *Bothrops* species (Brasil 2023). Three Brazilian institutes keep snakes in captivity to produce antivenom (i.e., Instituto Vital Brazil; Instituto Butantan; and Fundação Ezequiel Dias; Brasil 1905; Squaiella‐Baptistão et al. 2018). To ensure the efficiency of venom production and the health of snakes in captivity, it is imperative that these animals receive an adequate and balanced diet (Arbuckle 2010; Salobir et al. 2012).

Factors like age, venom composition, body and head size, sex, and geographical distribution influence the nutritional and energy demands of each species (Breno et al. 1990; Oftedal and Allen 1996; Moon et al. 2019). Poor feed management for snakes in captivity can lead to a decrease in weight and, consequently, to anorexia or death (Mitchell 2004). Therefore, the selection of suitable prey for snakes in captivity to avoid high mortality rates is extremely important and requires adequate resources, including type and size of prey and environment temperature and humidity control, because it directly impacts the maintenance, survival, growth, and reproduction of snakes in captivity (Bronikowski and Bronikowski 2000; Madsen and Shine 2002).

Research focusing on captive snakes' nutrition or dietary preferences is scarce (Oftedal and Allen 1996; Mitchell 2004; Arbuckle 2010; Salobir et al. 2012). Most studies use mice as food and focus on snakes' feeding behavior (Hamilton 1951; Scartozzoni and Molina 2004; Urdaneta et al. 2004). The use of day‐old chicks as food for captive snakes is also discussed (see more details in Arbuckle 2010), but no studies focusing on amphibians as food for an ontogenetic diet shift in snake species have been performed so far.

*Bothrops* snakes, including *B. jararacussu*, have a specialist diet with an ontogenetic shift (e.g., Sazima 1991; Marques et al. 2004; Caldart et al. 2011), where young predominantly feed on ectotherms, as anurans, whereas adults feed almost exclusively on endotherms, mainly rodents (Marques et al. 2001; Marques et al. 2004; Hartmann et al. 2009). In this sense, we tested whether exotic and native anurans can be an alternative to mice to feed young and adult individuals of *B. jararacussu* in captivity.

## 2 Materials and Methods

**Figure 1:**
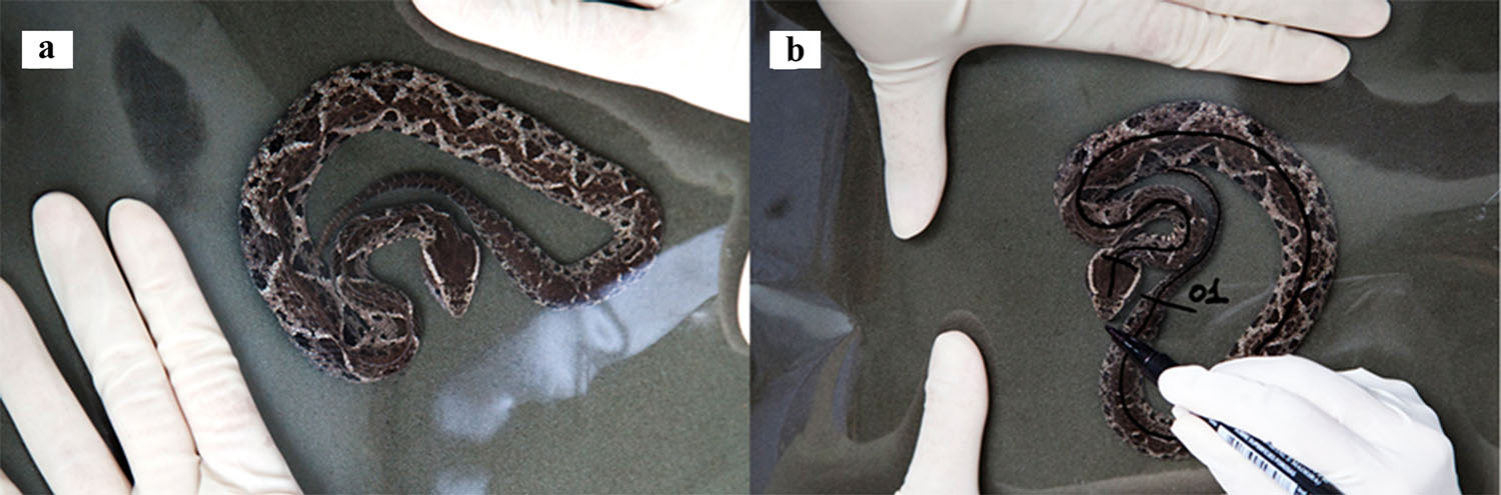
Methodological snake measurements. (a) *Bothrops jararacussu* juvenile containment between the acrylic sheet and the foam. (b) Line drawing from the snout to the tail base following the animal's spine, used for the measurement process.

We tested 41 individuals of *B. jararacussu* (17 young, 11 females and six males; and 23 adults, 13 females and ten males). The young and adult snakes belong to the pitvipers reproduction at the Division of Medical Zoology, Vital Brazil Institute (VBI). Adults are used for reproduction, while their young replace the snakes used for venom extraction and antivenom production. The number of individuals in our experiments followed the VBI sex and age range availability. We kept the snakes in a climate‐controlled room at 24°C to 26°C and 60% to 80% humidity in individual polypropylene boxes with adequate dimensions for the age: 30x20x12 cm (young) and 50x30x18 cm (adults). Each box contained corrugated cardboard and a water container. The animals' weights were measured using a Toledo electronic scale (0.001 g). The snakes' sizes were measured by immobilizing each snake between foam and a plastic sheet (acetate or acrylic), drawing a line along the snake's spine, and measuring the line with a flexible tape to the nearest cm (Figure 1a,b). The sex of each individual was determined via cloacal probing (Buononato 2018).

Feeding experiments ran from June 2012 to September 2012. The snakes were fed on tadpoles and adult bullfrogs (*Lithobates castebianus)*, sourced from Ranit Frog farm, Rio de Janeiro State, Brazil. The bullfrog is a commercialized species known for its ease of management, rapid growth, and high number of eggs per clutch.

We sourced five species of native Atlantic Forest hylids (*Boana albormarginata* (Spix, 1824), *Scinax hayii* (Barbour, 1909), *S. alter* (Lutz, 1973), *S. x‐signatus* (Spix, 1824), and *Ololygon angrensis* (Lutz, 1973) mainly from Reserva Rio das Pedras (RERP), in Mangaratiba municipality, and Jardim Botânico do Rio de Janeiro (JBRJ), Rio de Janeiro State. After collection, the anurans were kept in captivity and fed on cricket (*Gryllus* sp.) from VBI bioterium until offered as food for the snakes.

We partitioned snakes into five diet‐based groups using mice (*Mus musculus*) as the control group (Table 1). We offered food weekly for the young and biweekly for the adults for 3 months. The mice were dead, while the anurans (tadpoles and adults) were offered alive. Rodents are known to scratch and bite their predators in the wild, so in captivity, the offer of dead mice helps avoid a retaliation risk from the snakes. The prey's length was not measured, but we offered prey of similar sizes to the snakes. The weight of the anuran prey was comparable to that of mice offered (Table 1).

**Table 1:** Experimental protocols performed in the captive snake *Bothrops jararacussu* at the Vital Brazil Institute from June 2012 to September 2012. Abbreviations are: ♀ = Female, ♂ = Male, ANA = Adult native anurans, AB = Adult Bullfrog, M = Mice, and BT = Bullfrog tadpoles. Values of the weight column represent the mean + one standard deviation of the mean.

Group	*N* individuals	Age range	Sex		Food		
			♀	♂	Animals	Weight	Offer
1	9	Young	7	2	M	102,4 ± 37,4	Weekly
2	4	Young	3	1	BT	100,5 ± 36,9	Weekly
3	5	Young	1	4	ANA	101,7 ± 21,4	Weekly
4	12	Adult	7	5	M	118,0 ± 35,8	Biweekly
5	11	Adult	6	5	AB	115,9 ± 34,2	Biweekly

All snakes were weighed and measured at fixed intervals (monthly for young and every 2 months for adults) to monitor animal growth (total length ‐ TL) and weight gain (body mass ‐ BM) (Table 1 – Supplementary file). The individuals' sex was not considered for the statistical analysis due to the small sample size. We used a general linear model (GLM) to compare the change between TL and BM of each diet group. After the first GLM analysis, the residuals did not meet the criteria for normality and homoscedasticity, so the variables were log‐transformed, and a new analysis was performed. The results were analyzed statistically using the software R (R Core Team 2020) with the confidence level set at 5% (*p* < 0.05) (Zar 1999).

## 3 Results

**Figure 2:**
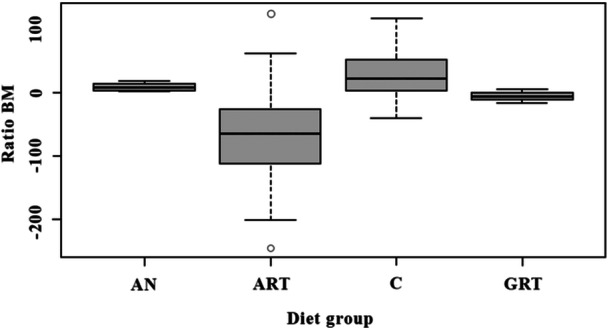
Body Mass ratio of each diet group of *Bothrops jararacussu*. AN = native anuran, ART = adult of bullfrog; C = mice, GRT = tadpole of bullfrog.

**Figure 3:**
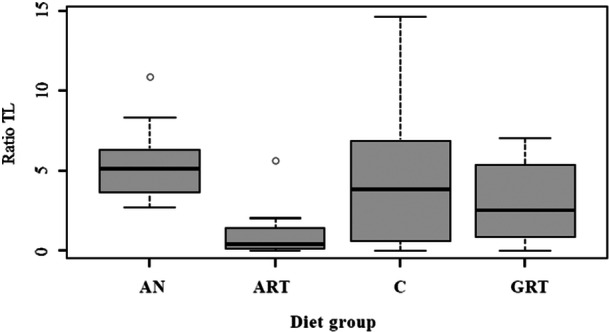
TL ratio of each diet group of *Bothrops jararacussu*. AN = native anuran, ART = adult of bullfrog, C = mice, GRT = tadpole of bullfrog.

**Figure 4:**
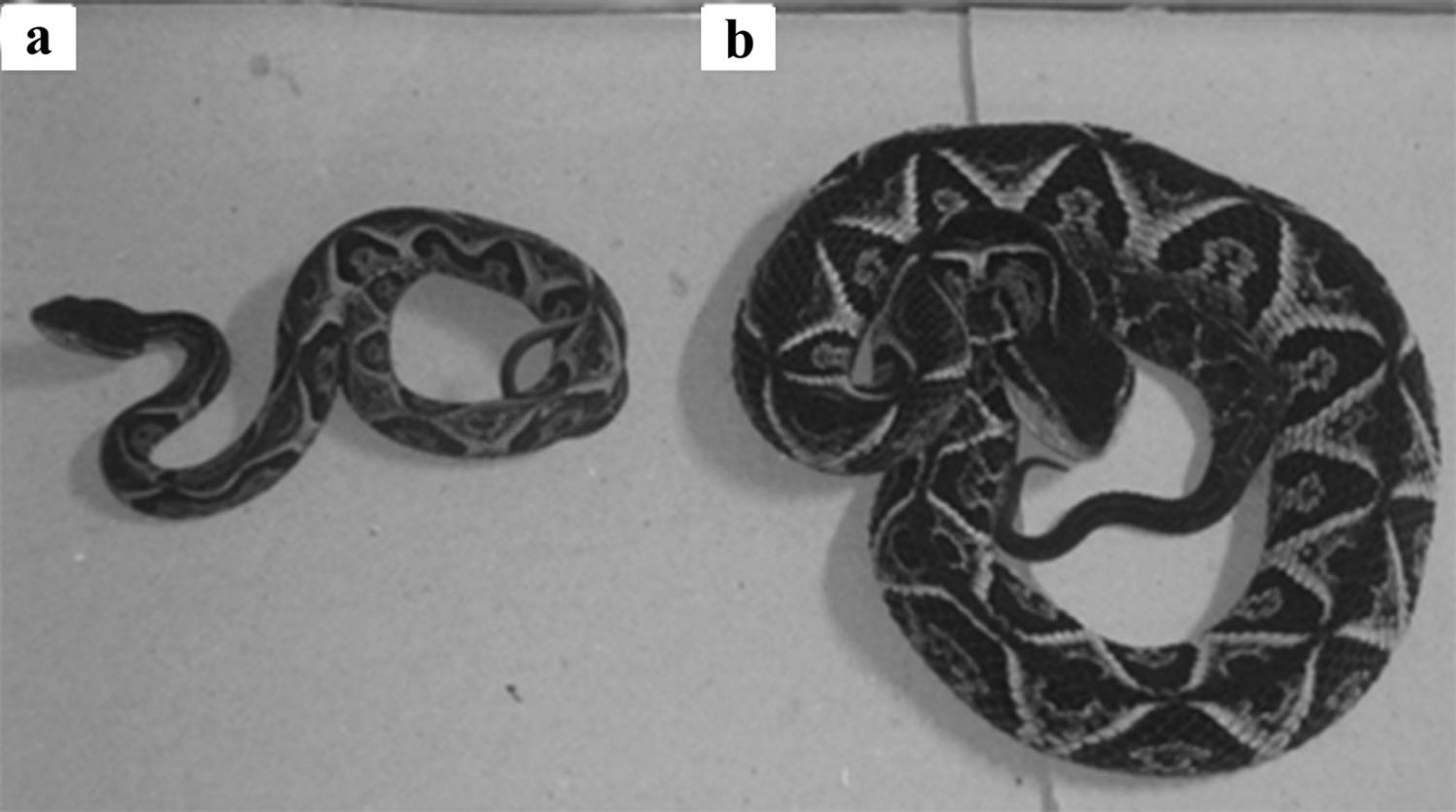
Difference between two *Bothrops jararacussu* young individuals feed on different prey. (a) bullfrog tadpole feed. (b) mice feed.

The GLM analysis revealed that regardless of the age group, *B. jararacussu* exhibited lower BM when fed with bullfrogs (adults *p* < 0.01 and tadpoles *p* < 0.01) compared to those provided with native anurans or mice (*p* ≥ 0.05) (Figure 2). The optimal model, incorporating TL of each diet group, demonstrated that young snakes fed native anurans (*p* < 0.001) had higher BM and consequently exhibited superior growth than those fed mice or bullfrogs (*p* ≥ 0.05) (Figures 3, 4).

**Figure 5:**
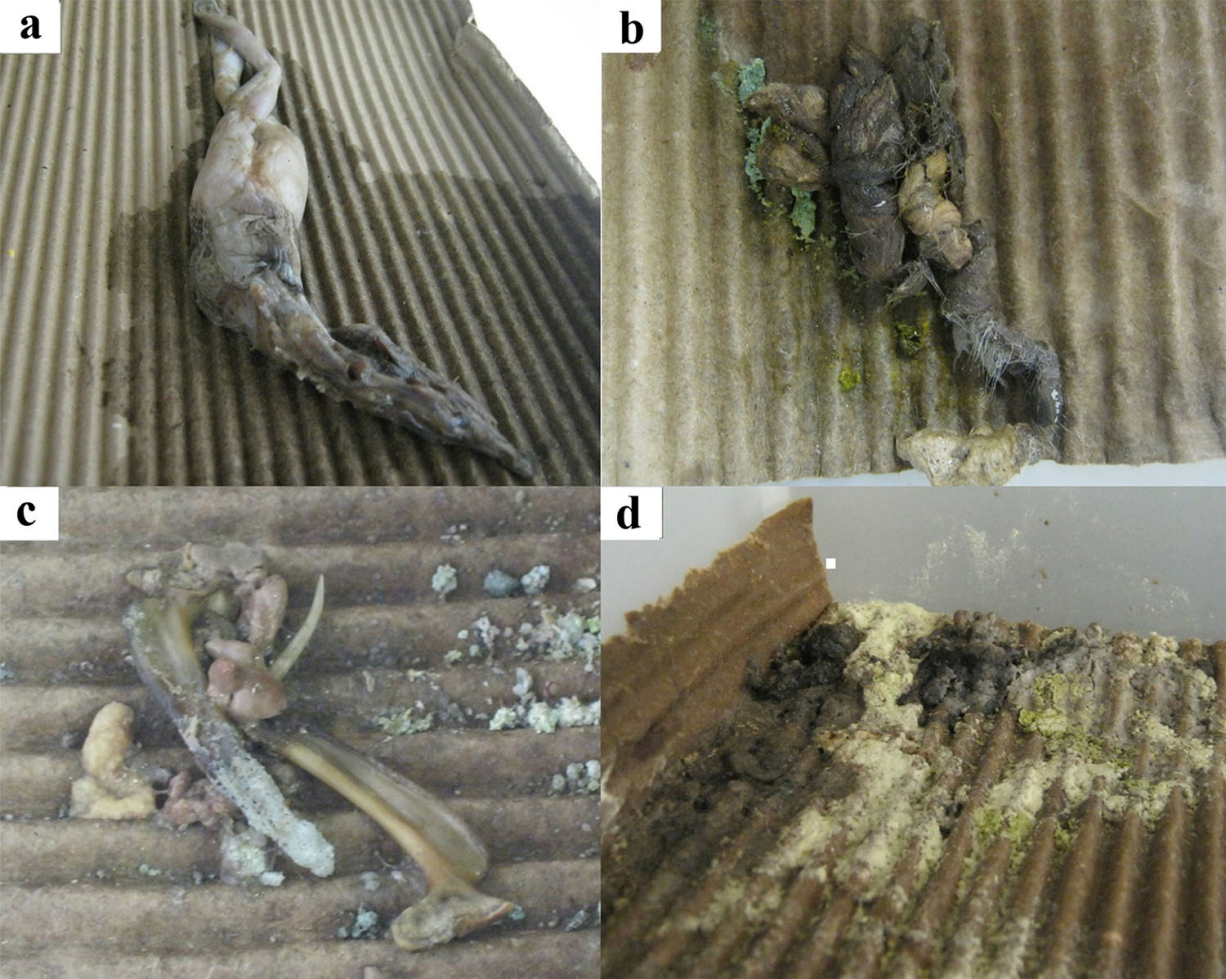
Atypical *Bothrops jararacussu* stools during the experiment. (a) Bullfrog regurgitated by a *B. jararacussu* adult. (b) Normal feces from *B. jararacussu* fed mice. (c) Bullfrog undigested bones in *Bothrops jararacussu* adult feces. (d) Abnormal feces from *B. jararacussu* fed bullfrogs.. [Correction added on 14^th^ May 2025, after first online publication: Descriptions for Figure 5 panels b and d have been corrected.]

During our experiments, juvenile individuals in group 2 exhibited several health issues, including weight loss, regurgitation, and the presence of atypical fecal matter. The prey in question was consequently withdrawn, and the mice were once again made available to avoid any adverse effects on the snakes' health. Despite our efforts, all juvenile snakes did not accept food offers and died (Table I – Supplementary file). Conversely, adult individuals in group 5 did not entirely refuse food but began to consume it less frequently (Figure 5). After 3 months, they began to regurgitate the prey, and we returned to offering mice to avoid health problems for them. In contrast to the juveniles, the adults accepted the return of mice to their diet without issue. No adult snakes died.

## 4 Discussion

Our findings suggest a positive correlation between TL gain and the groups consuming native anurans. On the other hand, BM exhibited significance across all age groups of *B. jararacussu* that consumed bullfrogs, indicating that snakes fed bullfrogs had lower body mass and consequently shorter total length compared to those in other dietary groups. Although young individuals of pit vipers feed on anurans in the wild (Almeida–Santos and Salomão 2002; Caldart et al. 2011), the low feed performance, weight loss, regurgitation, atypical feces, and death of some individuals fed with bullfrog tadpoles (Figure 5) indicate that this species is not an adequate food for *B. jararacussu* in captivity.

Regurgitation and atypical feces were also observed using bullfrog tadpoles to feed the black bass fish, *Micropterus salmoides* (Lacepede, 1802), indicating that bullfrog tadpoles present a possible digestive dysfunction for the black bass fish (Kruse and Francis 1977). We know that certain peptides found on the skin of bullfrogs have the potential to alter the composition of gram‐positive or gram‐negative bacteria (Xu et al. 2017), which may explain the digestive dysfunction in black bass fish. Furthermore, many anurans, including *L. castesbianus*, possess chemical substances acting as defenses against predators (e.g., Daly et al. 2005; Roelants et al. 2010; Wake and Koo 2018), which may explain the digestive dysfunction in black bass fish. Since we did not test this in our experiments, we propose that future studies explore the comparison between providing anurans with or without skin to snakes. Alternatively, researchers could delve into comprehending the impact of including *L. castesbianus* in the diet on snakes' metabolism.

During our experiments, snakes fed with native anurans did not refuse, regurgitate, or have atypical feces, indicating that such responses could be related to the bullfrog diet. In addition, most snakes in this group (95%, *n* = 4) bit and held the food offered, which is expected for *Bothrops* snakes when eating birds or frogs in the wild (Martins et al. 2002). Conversely, the growth observed in this dietary group could be attributed to the fact that adults of native anurans might contain more protein and fat per gram compared to bullfrog tadpoles. Even though some authors suggest that a mice diet can be more energetically profitable than feeding on ectothermic organisms (Kauffeld 1953; Mitchell 2004), no further studies have tested this hypothesis.

Our results suggest native anurans could be used as an alternative diet for *B. jararacussu* in captivity. We understand that using native species could represent a complex task due to the logistics of collecting and keeping these animals in captivity, considering their threatened status and abundance in nature. In this sense, we recommend that upcoming studies concentrate on identifying non‐threatened target species and assess whether their collection impacts natural populations. Only after conducting these studies can we determine whether it is feasible to breed a native species to feed young snakes in captivity.

Given that food adequacy and nutrition play crucial roles in the optimal growth, maintenance, health, and reproduction of captive snakes (Oftedal and Allen 1996; Madsen and Shine 2002), studies addressing and exploring dietary aspects of captive animals must continue to be conducted. While mice have commonly served as a staple food for captive snakes, it becomes essential to strike a balance between the ease of feeding and enhancing the nutritional quality of their diet (Mitchell 2004), aiming to provide prey that more closely resembles what they would consume in the wild. We anticipate that our data contribute with relevant information to future research, fostering improvements in developing projects involving endangered species in conservationist captivity, such as VBI. Moreover, we hope to stimulate further research to explore the feasibility of offering alternative prey items to snakes in captivity.

## Author Contributions

**Taís Vasques Torrents:** investigation, collected data, and methodology, **Fernanda Dias‐Silva:** investigation and writing, **Luisa Diele‐Viegas:** methodology, formal analysis and writing (review and edition), **Ana Maria Paulino Telles de Carvalho‐e‐Silva:** writing (review and edition) and supervision.

## Supporting information


**SUPPLEMENTARY FILE.** Table 1: The size and mass of each experimental snake. BM= body mass; TL= total length; t_0_=measure before start the experiment; t_3_= last measure of experiment; *=Individual came dead.

## Data Availability

The data supporting the findings of this study are available from the corresponding author upon request.
