# Supplementary material for: Anurans or Mice: What is the Best Food Item for Young and Adults of Bothrops jararacussu (Lacerda, 1884) in Captivity?
Source: Zoo Biol. 2025 May 3;44(4):371–6. doi: 10.1002/zoo.21904 (PMC12335228; doi:10.1002/zoo.21904)
Supplement: Supplementary file 1 — SUPPLEMENTARY FILE. Table 1: The size and mass of each experimental snake. BM= body mass; TL= total length; t0=measure before start the experiment; t3= last measure of experiment; *=Individual came dead. [file ZOO-44-371-s001.docx]

**SUPPLEMENTARY FILE**

Table 1: The size and mass of each experimental snake. BM= body mass; TL= total length; t_0_=measure before start the experiment; t_3_= last measure of experiment; *=Individual came dead.

| **N individual** | **Group** | **BM** | | **TL** | |
| --- | --- | --- | --- | --- | --- |
|  |  | **t_0_** | **t_3_** | **t_0_** | **t_3_** |
| 16295 | 1 | 36 | 82 | 14,2 | 31 |
| 16300 | 1 | 30 | 68 | 13,4 | 24,7 |
| 16302 | 1 | 48 | 80 | 22,9 | 38,7 |
| 16304 | 1 | 52 | 80 | 22,7 | 34,1 |
| 17420 | 1 | 30 | 42 | 32,7 | 47,5 |
| 17421 | 1 | 30 | 54 | 30,9 | 48,2 |
| 17422 | 1 | 34 | 58 | 32 | 50,5 |
| 17429 | 1 | 32 | 50 | 29,6 | 46,3 |
| 17430 | 1 | 36 | 56 | 33,5 | 56,6 |
| 16298 | 2 | 34 | 34 | 15,2 | 21,7 |
| 16299* | 2 | 40 | 30 | 18,6 | 23,4 |
| 16301* | 2 | 42 | 34 | 18,3 | 26,8 |
| 16303 | 2 | 46 | 34 | 28,9 | 31,6 |
| 17423 | 3 | 34 | 56 | 32,3 | 53,5 |
| 17427 | 3 | 30 | 44 | 31,7 | 50,6 |
| 17428 | 3 | 30 | 44 | 29,1 | 45,6 |
| 17431 | 3 | 32 | 40 | 30,2 | 49,1 |
| 17433 | 3 | 32 | 48 | 29 | 49,5 |
| 13404 | 4 | 344 | 356 | 101,7 | 102 |
| 14320 | 4 | 662 | 700 | 111,5 | 115,9 |
| 14324 | 4 | 362 | 410 | 102,7 | 104,2 |
| 14330 | 4 | 654 | 706 | 113,2 | 114,1 |
| 14334 | 4 | 660 | 650 | 113,3 | 117,9 |
| 14336 | 4 | 868 | 950 | 116,8 | 117,7 |
| 14344 | 4 | 762 | 808 | 117,1 | 119,7 |
| 14347 | 4 | 438 | 508 | 100,5 | 101,1 |
| 14348 | 4 | 428 | 418 | 111,9 | 111,9 |
| 14355 | 4 | 754 | 832 | 118,2 | 120 |
| 14356 | 4 | 276 | 278 | 100,1 | 100,4 |
| 14362 | 4 | 908 | 904 | 127 | 132,7 |
| 14328 | 5 | 470 | 268 | 123,3 | 124 |
| 14335 | 5 | 260 | 234 | 92,2 | 93,3 |
| 14337 | 5 | 368 | 284 | 103,6 | 105,9 |
| 14339 | 5 | 416 | 364 | 116,1 | 121,9 |
| 14340 | 5 | 378 | 326 | 107,6 | 108,2 |
| 14341 | 5 | 456 | 368 | 110,8 | 111,8 |
| 14342 | 5 | 344 | 328 | 104,7 | 105,7 |
| 14345 | 5 | 404 | 394 | 111,4 | 111,7 |
| 14349 | 5 | 344 | 380 | 101,7 | 103,8 |
| 14350 | 5 | 292 | 266 | 107 | 107,6 |
| 14353 | 5 | 284 | 264 | 105,1 | 106,3 |
